# Hormonal Male Contraception: Getting to Market

**DOI:** 10.3389/fendo.2022.891589

**Published:** 2022-06-03

**Authors:** Stephanie T. Page, Diana Blithe, Christina Wang

**Affiliations:** ^1^ Division of Metabolism, Endocrinology and Nutrition, University of Washington, Seattle, WA, United States; ^2^ Contraceptive Development Program, Eunice Kennedy Shriver National Institute of Child Health and Human Development, National Institutes of Health (NIH), Bethesda, MD, United States; ^3^ Clinical and Translational Science Institute at the Lundquist Institute at Harbor-UCLA Medical Center, Torrance, CA, United States

**Keywords:** androgen, testosterone, sperm, male contraception, male contraception emerging market, population growth, acceptability

## Abstract

Rates of unplanned pregnancies are high and stagnant globally, burdening women, families and the environment. Local limitations placed upon contraceptive access and abortion services exacerbate global disparities for women. Despite survey data suggesting men and their partners are eager for expanded male contraceptive options, efforts to develop such agents have been stymied by a paucity of monetary investment. Modern male hormonal contraception, like female hormonal methods, relies upon exogenous progestins to suppress the hypothalamic-pituitary-gonadal axis, in turn suppressing testicular testosterone production and sperm maturation. Addition of an androgen augments gonadotropin suppression, more effectively suppressing spermatogenesis in men, and provides androgenic support for male physiology. Previous contraceptive efficacy studies in couples have shown that hormonal male methods are effective and reversible. Recent efforts have been directed at addressing potential user and regulatory concerns by utilizing novel steroids and varied routes of hormone delivery. Provision of effective contraceptive options for men and women is an urgent public health need. Recognizing and addressing the gaps in our contraceptive options and engaging men in family planning will help reduce rates of unplanned pregnancies in the coming decades.

## Introduction

Globally, unplanned pregnancy rates have remained high over the last three decades, a time that has coincided with global warming, population growth and increasing calls for policies that decrease greenhouse gas emissions (1). Limited access, education and engagement in modern, effective contraception remains a global problem that disempowers women, contributes to a cycle of poverty, and impacts the health and welfare of girls and adolescents. However, even with increased access, many women experience side effects from currently available contraceptives or have health conditions that limit contraceptive use. While 16% (6% Africa, 29% North America) of current global contraceptive use is male-directed (condoms, vasectomy and withdrawal) (2), male engagement in contraception is variable around the globe and stymied by limited choices and high rates of method failure (condoms, withdrawal). Vasectomy is effective but requires a highly skilled provider, is invasive and largely irreversible, limiting the population willing to use this method. We are overdue for new options for male contraception, including both reversible and permanent methods.

Despite the imperatives of climate change and population growth, investment in development of novel, reversible contraceptives for men is minimal, compared with the late 20^th^ century when academia, non-governmental organizations, pharmaceutical entities, and governmental agencies were investors. In the last decade, pharmaceutical companies have largely abandoned their male contraceptive development programs. Can this trend be reversed? We believe we are at a turning point with new male contraceptive methodologies showing promise and strongly positive receptivity from both men and their partners (3–6). It is time for a sea change in investment in male contraception, a potential game-changer for family planning, female agency and reproductive rights.

## Is There a Market for Novel Male Contraceptives?

Conceptually, male contraceptives, including hormonal male methods, appear to have high acceptability amongst potential users. Multinational survey data from the early 2000s suggest interest and enthusiasm among men from a variety of countries (3–6). Women in committed relationships state they are likely to trust their partners to use these methods (5) and demand for these methods is likely to grow with increasing public awareness. Data obtained from participants in male contraceptive clinical trials represent real user experience, albeit self-selected volunteers with baseline willingness to engage in male contraceptive development (7–10). Across multiple studies employing various modes of administration, participants are overwhelmingly positive regarding the products evaluated, with 50-85% of men reporting willingness to use the product and pay out-of-pocket if commercially available.

Creative methods to demonstrate user desires and preferences are needed to harness the interest of the pharmaceutical industry to support male contraceptive development. Landscape surveys of potential users in least-resourced regions to assess men’s willingness to share the burden and costs for contraception are necessary to advance the funding, development, and marketing of new male contraceptive methods. A non-profit (Male Contraceptive Initiative) committed to helping develop male contraceptives has recently conducted consumer market research among men in the United States. Their findings echo earlier enthusiasm; approximately 50% of US men, ages 18-49 who have sex with women and do not wish to father a pregnancy express a high level of interest in novel male contraceptives (11). Data have long suggested that male attitudes toward family planning, including child spacing and family size, have a strong influence on contraceptive use by women and within a couple (12), including in Africa and Southeast Asia where the global burden of maternal death is the highest (WHO trends in maternal mortality). Indeed, pilot projects in sub-Saharan Africa, such as the Malawi Male Motivation project, demonstrate that contraceptive education programs aimed at men improve contraceptive uptake and communication around sexual health within couples, even among couples who have never used contraception (13). As men become more engaged in reproductive health, updated work is needed to better understand the modern contraceptive landscape.

For effective uptake, novel contraceptives must be cost-effective for users and for public health programs aimed to assist family planning. In this context, it is noteworthy that in the United States, long-acting reversible contraceptives (LARCs) female intrauterine devices and implants, are the most cost-effective contraceptive methods, despite high up-front costs (14). Modelling predicts that introduction of novel, reversible male methods could significantly decrease unintended pregnancies as much as 30-40%, particularly in areas where contraceptive uptake is currently low (15). The toll of unplanned pregnancies, as well as medical abortions, is high, affecting mental, physical and economic well-being of women and families. Data on immediate health care costs alone support significant cost-effectiveness for increased contraceptive use among high-risk populations (16). Introduction of novel, cost-effective male contraceptives could have important downstream global health and economic benefits that, over time, could decrease health disparities.

## Hormonal Male Contraception Is Effective and Reversible

Like hormonal female methods, hormonal male contraceptives utilize exogenous steroids to interrupt physiologic hypothalamic-pituitary-gonadal pathways. Exogenous steroids suppress secretion of gonadotropins, LH and FSH; lack of gonadotropins impairs testosterone production and sperm maturation in the testes, resulting in profound reductions in sperm output 4-12 weeks following initiation of the method. Proof-of-principle studies in the 1980s performed by the World Health Organization (WHO) demonstrated that exogenous high-dose androgens given to healthy men markedly, and reversibly, suppressed spermatogenesis and provided effective contraception for couples (17–19). The use of exogenous progestins more profoundly suppresses gonadotropin secretion in men and allows for physiologic dosing of androgens, largely eliminating hyper-androgenic side effects and minimizing time to suppression to effective contraceptive thresholds (<1 million sperm/ml of ejaculate) (20).

To date, a total of eight hormonal male contraceptive efficacy studies have been conducted, five utilized only testosterone derivatives and three administered a progestin plus testosterone (9, 18, 21–26). Over 2000 couples have been enrolled in these trials, with >1500 completing the efficacy phase (wherein the study drug is relied upon as the sole contraceptive method) after achieving a predetermined sperm threshold of <1-5 million sperm/ml. The Pearl Index, a measure of failure rate, has ranged from 0-2.3 pregnancies/100 person-years in male hormonal contraceptive clinical trial when a sperm threshold of < 1 million/ml. This compares favorably with female hormonal methods ranging from 0 to 0.3 for intrauterine devices and implants to 1 to 3% for perfect use of the oral contraceptive pill. However, the typical failure rate for female injectable contraceptives is estimated as 6%, for female oral contraceptives is 7.2% and for male condoms is 13%. Whilst regulators have yet to provide firm guidance regarding acceptable failure rates for novel male contraceptives, investigators have advocated for approval of new male methods that fall in the typical use range of condoms. In all studies of these male contraceptive regimens, the methods were fully reversible (27). Thus, although data are limited to the clinical trial context, hormonal male contraceptive methods are highly effective.

## What Is in The Male Contraceptive Clinical Pipeline?

Hormonal male contraceptive trials over the last five decades have largely centered upon longer-acting hormonal therapies administered by a clinician (i.e. implants, intramuscular injections). With the approval of transdermal formulations of testosterone, research supported by the *Eunice Shriver Kennedy* National Institute of Child Health and Human Development (NICHD) in collaboration with the Population Council, has evaluated transdermal gels delivering a novel progestin, segesterone acetate (also known as Nestorone^®^) and testosterone to inhibit sperm production. This transdermal NES/T gel has the potential to provide more independence and less discomfort for users than injections and implants and has few side effects whilst delivering physiologic doses of androgens (28–31).

We are conducting a Phase 2b contraceptive efficacy study of NES/T transdermal gel. This multi-national study enrolling 400 couples is the first to evaluate contraceptive efficacy of a daily, self-delivered male contraceptive agent. Importantly, with sites in the United States, Europe, South America and Africa, it will provide information from a diverse group of potential users and is the first male contraceptive efficacy study to include a site in Sub-Saharan Africa. Early clinical studies of NES/T gel demonstrated high effectiveness at suppressing gonadotropins and sperm production (29–31), and very high acceptability amongst users (32) who were eager to know when this product will be commercially available for male contraception. Clinical evaluation of the potential for transfer of the transdermal hormones to a partner was reassuring when the gel was used as instructed (33). Most men found the product easy to use and they adapted the daily gel application to their routine grooming. Results to date indicate that the product is highly effective and acceptable to both partners. Large Phase 3 pivotal studies to further demonstrate safety and contraceptive efficacy are needed for regulatory approval and will require involvement of the pharmaceutical industry.

A notable deficiency in hormonal male contraceptive development and clinical testing has been candidate oral formulations. Many men report they would prefer an oral agent to other modes of contraceptive delivery (3). Until recently, approved oral testosterone formulations have been associated with hepatoxicity (methyltestosterone). A recently approved oral formulation of T undecanoate (34) is safe but the requirement for twice-daily dosing with food is not convenient for a contraceptive regimen. To fill this gap, NICHD is developing several novel androgens as oral formulations in an effort to design the elusive “male pill”.

Dimethandrolone undecanoate (7-alpha, 11-beta-dimethyl-19-nortestosterone undecanoate (DMAU)) and 11-beta-methyl-19-Nortestosterone 17-beta-dodecylcarbonate (11β-MNTDC), are synthetic pro-drugs under investigation as both oral and injectable contraceptive agents. DMAU is converted to the active drug, DMA, and 11β-MNTDC to 11β-MNT, *in vivo*, by endogenous esterases. DMA and 11β-MNT activate both androgen and progesterone receptors (35). These progestogenic androgens have potential to be single-agent male hormonal contraceptives. Neither androgen requires 5alpha-reduction (36) to exert maximal androgenic action and neither is aromatized to an aromatic A-ring compound (37). *In vitro*, DMAU is a more potent androgen, while 11β-MNTDC has more balanced androgen and progestogenic activity (35, 38); thus, they exhibit slightly different pharmacodynamics in men.

Preclinical studies in rodents demonstrated that DMAU reversibly suppressed gonadotropins, spermatogenesis and fertility while maintaining non-gonadal androgenic effects (39–41). Both DMAU and 11β-MNTDC support androgenic body composition and bone mineral density in mice (39). Initial studies of single oral doses of DMAU and 11β-MNTDC in men demonstrated that concomitant food ingestion is required for effective oral absorption of these synthetic steroids (42, 43). A subsequent dose-finding study in healthy men,100-400 mg of DMAU taken once-daily for 28 days, provided further evidence that oral DMAU is safe, well-tolerated and markedly suppressed serum gonadotropins and sex-steroid concentrations (44). Remarkably, participants receiving DMAU rapidly developed castrate serum testosterone concentrations (<50 ng/dL), yet had few or no symptoms of hypogonadism, a profound *in vivo* demonstration of the androgenic activity of DMA previously observed *in vitro* (35). A longer study of daily oral DMAU, 100-400 mg, to determine its impact on spermatogenesis is underway. A Phase 1 study of DMAU as an injectable male contraceptive is also underway (NCT02927210). Intramuscular administration of DMAU is unlikely to cause changes in serum lipids by avoiding the well-recognized first-pass effects of oral androgen administration on cholesterol metabolism (45) and may act as a “depot” formulation allowing for injection intervals of 2-6 months.

Compared to other androgens, 11β-MNTDC is the least hepatotoxic when given in repeated oral doses to rabbits (40), making it a promising oral agent. Like DMAU, a 28-day daily dosing study of 200- 400 mg doses of 11βMNTDC demonstrated profound suppression of serum testosterone and gonadotropins, particularly at the higher dose (46). Side effects noted were mild and similar to DMAU. Longer studies of these dual-action androgens are ongoing to determine their relative potency as reversible inhibitors of spermatogenesis; they show considerable promise as a male pill.

Non-hormonal approaches to reversible male contraception aim to reversibly disrupt testes or germ-cell specific targets. These targets include structures and molecules involved in sperm transport, spermiation, and sperm motility among others. A recent review of these approaches has been published (47); major hurdles in advancing development of non-hormonal contraception for men includes ensuring specificity, drugability, safety, and efficacy in animal models. With the exception of trials in India of reversible vaso-occlusion (48), where reversibility remains a major challenge, novel non-hormonal contraceptives for men have not reached clinical trials. It is likely that hormonal male contraceptives will be the first novel, reversible male method to reach the marketplace, hopefully paving the way for additional methods to contribute to the male contraceptive menu going forward.

## Risks and Benefits of Hormonal Male Contraceptives

Similar to female hormonal contraceptives, some men who use investigational hormonal male contraceptives may experience unwanted side effects. In general, side effects are seen in a minority of men and mirror those experienced and tolerated by, women who use hormonal methods; namely mild weight gain, mood lability, and impacts on libido. Early hormonal male contraceptive efficacy studies utilized supraphysiologic dosages of intramuscular testosterone. Reported androgenic side effects in some participants, included significant increases in hematocrit, creatinine, and triglycerides across the studies (17–19). Utilizing progestins facilitates physiologic androgen dosing side effects were minimal in recent male contraceptive efficacy studies (22) (21). Pre-efficacy studies in male volunteers demonstrate that the frequency and severity of side effects may be impacted by the progestin used (and its relative androgenicity) and the mode of administration. For example, a series of studies combining oral levonorgestrel with physiologic doses of intramuscular testosterone demonstrated that reductions in levonorgestrel dosing minimized side effects such as weight gain without impacting sperm suppression (49–51). Transdermal delivery of Nestorone^®^/Testosterone gel is well-tolerated. The most common side effects some men experience are modest weight gain (2-5kg) and acne (30). Fine-tuning the dose of testosterone and the progestin may minimize some side effects that were observed in earlier studies. Nestorone is a potent progestin, that may have advantages over other progestins; its lack of cross reactivity with androgen and estrogen receptors may limit side effects (28).

Validated tools to prospectively identify and quantify potential impacts on mood, libido and sexual function are critical to include in all placebo-controlled Phase 1 and Phase 2 male contraceptive studies, as well as in future efficacy trials, to better understand possible effects of exogenous steroids on these health parameters in men. A placebo-controlled sperm suppression study using a long-acting progestin implant and long-acting TU injections for T replacement, highlighted that male hormonal contraceptives might have mood-related side effects in some men (52). A subsequent efficacy study of separate injections of a long-acting progestin and TU was prematurely terminated due to similar concerns (9). The potential for hormonal imbalance of progestin and testosterone with long-acting formulations may explain the mood effects observed in these trials. Studies of Nestorone and Testosterone combined in a gel and applied once-a-day have been reassuring to date, with no indication of changes in mood. Mild changes in libido without impact on sexual function were observed in a small minority of participants (30). Importantly, most participants report high satisfaction and both partners express a desire to continue to use this method, suggesting that side effects are minimal and acceptable to most users (29, 32). DMAU and 11β-MNTDC, despite leading to marked suppression of endogenous testosterone, were able to maintain sexual function with minor anticipated effects on hematocrit and lipid profiles (53–55). In short-term studies, participants found the once–a-day oral capsules highly acceptable (56, 57).

The risk/benefit ratio for male contraception is complex. Women weigh the side effects of contraceptive methods relative to effects of an unwanted pregnancy; however, the personal risk/benefit health ratio is different for men, raising questions regarding their tolerance for potential side effects. Ideally, male methods that have positive health benefits for the user (such as reductions in long-term disease risk, improvements in well-being, improved metabolic or skeletal risk profiles) will be identified, similar to benefits of some female hormonal methods. While men do not experience medical risks of pregnancy, exploring the mental and economic costs and benefits men *and their partners* incur with unwanted pregnancy will be important to quantify as we assess the potential impact of any novel male contraceptive. Indeed, the concept of “shared risk” is not novel in healthcare, and the importance of applying this principle to male contraceptives that provide substantial benefits to women and men must not be overlooked (58).

## Is There a Path to the Market for Novel Male Contraceptives?

A major impediment to moving male contraceptive development forward is a lack of regulatory guidance, inhibiting both scientific and financial investment. While work is ongoing to develop effective and well-tolerated products, it is not known what regulatory agencies such as the US Food and Drug Administration (FDA) and the European Medicines Agency (EMA) will find permissible for initial approval of the first hormonal male contraceptive. Consensus recommendations from the research community have been published (59) but whether these will be adopted by regulators is unknown

Along with scientific and clinical investment and innovation, behavioral studies to understand and address the impact of user variables, including product preferences, compliance, barriers to uptake, social biases, and access to contraceptives are critical to advancing the field of male contraception. Male-directed contraception is not new, but the last novel method, the condom, was introduced to the marketplace over 200 years ago. Novel male contraceptive methodologies demonstrate strongly positive receptivity from both men and their partners. Innovative experimental designs are needed to understand behavioral aspects of modern male contraceptive use. In parallel, engaging reproductive-age male and female advocacy groups will be critical to disseminating accurate information regarding novel male contraceptive methods, helping to reduce misinformation and disparities in access to products. Engaging pharmaceutical companies to co-develop products and initiate new pathways to product development is critical to moving the field forward. Lastly, fair pricing, prescribing practices and health care coverage will be necessary to ensure male contraceptives impact unplanned pregnancies and the global health of future generations of men and women.

## Data Availability Statement

The original contributions presented in the study are included in the article/supplementary material. Further inquiries can be directed to the corresponding author.

## Author Contributions

All authors (SP, DB, and CW) contributed to the concept, writing and editing of this manuscript. All authors read and approved the final manuscript.

## Funding

SP receives funding from the National Institutes of Health through the *Eunice Kennedy Shriver* National Institute of Child Health and Human Development for Clinical Evaluation of Male Contraceptives (Contract number: HHSN275201300025I). SP is also supported by the Robert McMillen Professorship in Lipid Research. CW receives funding form the *Eunice Kennedy Shriver* National Institute of Child Health and Human Development for Clinical Evaluation of Male Contraceptives: Contract number HHSN275220130024I and 75N94020D0007 and P50 HD098593 National Male Reprodutive Epigenomics Center and the National Center for Advancing Translational Sciences UL1TR 001881 UCLA Clinical and Translational Science Institute.

## Conflict of Interest

The authors declare that the research was conducted in the absence of any commercial or financial relationships that could be construed as a potential conflict of interest.

## Publisher’s Note

All claims expressed in this article are solely those of the authors and do not necessarily represent those of their affiliated organizations, or those of the publisher, the editors and the reviewers. Any product that may be evaluated in this article, or claim that may be made by its manufacturer, is not guaranteed or endorsed by the publisher.
